# Contradictions and conflict: A meta-ethnographic study of migrant women’s experiences of breastfeeding in a new country

**DOI:** 10.1186/1471-2393-12-163

**Published:** 2012-12-27

**Authors:** Virginia Schmied, Hannah Olley, Elaine Burns, Margie Duff, Cindy-Lee Dennis, Hannah G Dahlen

**Affiliations:** 1 School of Nursing and Midwifery, Family and Community Health Research Group, Locked Bag 1797, Penrith South DC, NSW, Australia; 2 Department of Psychiatry, Canada Research Chair in Perinatal Community Health, Shirley Brown Chair in Women's Mental Health Research, Women's College Research Institute, University of Toronto Lawrence S. Bloomberg Faculty of Nursing, 155 College St, Toronto, ON, M5T 1P8, Canada

**Keywords:** Meta-ethnography, Breastfeeding, Migrant, Immigrant, Refugee, Colostrum, Qualitative research

## Abstract

**Background:**

Studies report mixed findings about rates of both exclusive and partial breastfeeding amongst women who are migrants or refugees in high income countries. It is important to understand the beliefs and experiences that impact on migrant and refugee women’s infant feeding decisions in order to appropriately support women to breastfeed in a new country. The aim of this paper is to report the findings of a meta-ethnographic study that explored migrant and refugee women’s experiences and practices related to breastfeeding in a new country.

**Methods:**

CINAHL, MEDLINE, PubMed, SCOPUS and the Cochrane Library with Full Text databases were searched for the period January 2000 to May 2012. Out of 2355 papers retrieved 11 met the inclusion criteria. A meta-ethnographic synthesis was undertaken using the analytic strategies and theme synthesis techniques of reciprocal translation and refutational investigation. Quality appraisal was undertaken using the Critical Appraisal Skills Programme (CASP) tool.

**Results:**

Eight qualitative studies and three studies reporting both qualitative and quantitative data were included and one overarching theme emerged: ‘Breastfeeding in a new country: facing contradictions and conflict’. This theme comprised four sub-themes ‘Mother’s milk is best’; ‘Contradictions and conflict in breastfeeding practices’; ‘Producing breast milk requires energy and good health’; and ‘The dominant role of female relatives’. Migrant women who valued, but did not have access to, traditional postpartum practices, were more likely to cease breastfeeding. Women reported a clash between their individual beliefs and practices and the dominant practices in the new country, and also a tension with family members either in the country of origin or in the new country.

**Conclusion:**

Migrant women experience tensions in their breastfeeding experience and require support from professionals who can sensitively address their individual needs. Strategies to engage grandmothers in educational opportunities may offer a novel approach to breastfeeding support.

## Background

The World Health Organisation [1] recommends exclusive breastfeeding to six months however, exclusive breastfeeding rates in many high income countries decline dramatically following birth and at six months are reported as being less than 20% [2-4]. Reports vary on the rates of both exclusive and partial breastfeeding amongst women born in low to middle income countries who have migrated or are refugees in high income countries. Increased initiation and duration is reported amongst recent migrant women when compared to women born in the host country, particularly in countries such as United States and United Kingdom [5-7] but also in European countries [8,9]. However, this is not a consistent finding and there are reports of lower rates of initiation, duration and exclusivity amongst groups of migrant women such as Chinese and Vietnamese [9], most noticeable in countries with higher breastfeeding initiation rates such as Australia [10-13]. There is also increasing evidence that as women acculturate to the host country, they are more likely to take on breastfeeding practices that are comparable with those in the host country [6,14,15]. Studies report that for each year a migrant mother or father resides in a new country the duration of breastfeeding decreases [15-19].

Cross sectional surveys [15,20,21] and prospective cohort studies [5,22] have identified factors associated with breastfeeding initiation and duration amongst recent migrant and refugee women. These studies report similar barriers to breastfeeding as reported by non-migrant women, such as returning to work [5,17,23-25], pain [12,14,25,26], and perceived low breast milk supply [23-25,27-29]. Traditional postpartum beliefs and practices are also commonly reported as influencing continued breastfeeding to six months after birth [13,30,31].

Studies report that health services and breastfeeding support are not always accessible or culturally appropriate to meet the needs of migrant and refugee women [13,27,32,33]. Recent research by McFadden, Renfrew and Atkin [34] and Puthussery et al. [35] in the UK, indicates that maternity services often lack cultural sensitivity and health professionals have a tendency to make assumptions about, or stereotype, women from ethnic minorities. McFadden, Renfrew and Atkin particularly emphasised the tendency for health professionals to view ethnic minorities as homogenous groups, rather than diverse individuals, with a variety of cultural practices [34].

Well designed qualitative research describing both the similarities and diversity of migrant and refugee women’s beliefs and practices around breastfeeding can inform health service policy and practice and challenge common assumptions and stereotypes. In the last decade, there has been an increase in published qualitative studies exploring the beliefs and practices of migrant women related to breastfeeding and infant feeding decisions. This provides an opportunity to synthesise comparable studies in order to gain a broader understanding of the diversity of women’s experience of breastfeeding in a new country, and identify ways in which support provided to migrant and refugee women can be improved.

### Aim

The aim of this meta-ethnographic study was to explore the diversity of migrant and refugee women’s experiences and practices related to breastfeeding in a new country with a view to informing health care policy and practice and identifying ways in which migrant and refugee women can be better supported to achieve their breastfeeding goals.

## Methods

A meta-synthesis approach was undertaken using the analytic strategies and theme synthesis techniques of meta-ethnography including reciprocal translation and refutational investigation. Constant comparison and interpretation of reported qualitative findings can lead to a greater depth of understanding about the area of interest. Noblit and Hare [36] coined the term ‘meta-ethnography’ in 1988 and described this as a process involving the ‘translation’ of findings of one ethnographic study into the findings of the next to derive interpretive explanations and understanding. This approach has been developed further by other scholars [37,38] and is increasingly advocated as a technique that accompanies meta-analysis of interventions to inform health service policy and delivery [39]. In this paper we have synthesised multiple studies which have adopted a variety of methods; for example, qualitative description, ethnography and critical theory. Walsh and Downe [38] and Sandelowski [37] have argued that synthesising studies that utilise different qualitative methodologies achieves cross comparison of studies and facilitates the identification of similarities and differences in findings. The synthesising of qualitative findings does not amount to simply summing up or reducing the findings to one common conclusion, but rather is about enlarging the interpretive possibilities of findings [37].

### Search strategy

The literature search was conducted between September and November 2011 and revised in May 2012 using the following databases: CINAHL, MEDLINE, PubMed, SCOPUS. The Cochrane Library search terms included: migrant, immigrant, Non English Speaking Background, ethnic, ethnicity, refugee, recent arrivals, breastfeeding, infant feeding, postnatal and postpartum. Inclusion criteria were: a primary focus on the breastfeeding experiences and/or practices of migrant or refugee women. The term migrant can be ambiguous [40,41]. In this review, the term migrant was defined as women born in low or middle income countries, who had migrated permanently to a high income host country. Papers that included both migrant women and those born in the host country, including women from minority ethnic groups, were retained as long as the authors had identified the source of the data they reported. Studies were limited to those published in English between January 2000 and May 2012. While the prime focus of a meta-ethnographic study is on synthesising qualitative studies, we also included studies that reported both quantitative and qualitative data if a subset of the larger sample were interviewed or participated in focus groups yielding rich qualitative data. As not all databases enabled the use of search limiters, articles not satisfying these criteria were also removed during the first exclusion.

### Search results

**Figure 1:**
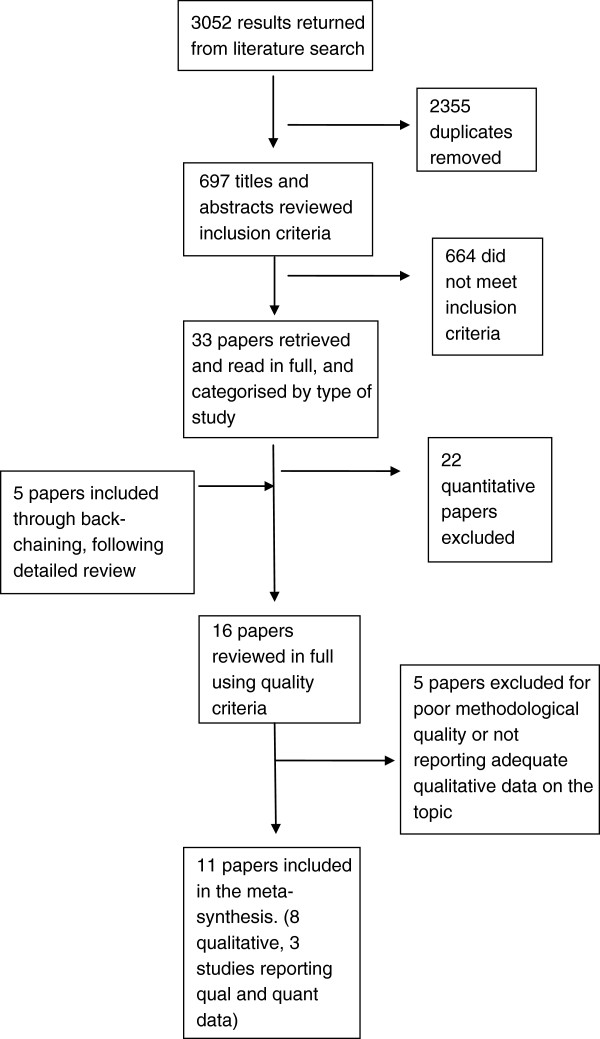
Search strategy and outcomes.

The search resulted in 3052 papers. Following removal of duplicates (2355 papers), 697 papers were reviewed by title and abstract to determine if they met the inclusion criteria. Thirty-three papers were identified and read in full. Twenty-two papers were then excluded as they did not meet inclusion criteria. Through ‘back-chaining’ [38], we identified and reviewed 10 additional papers, with five appropriate for full review (see Figure 1). Sixteen papers were included in the review.

### Data quality

Quality appraisal was undertaken using the Critical Appraisal Skills Programme (CASP) tool for qualitative research [42]. Sixteen papers were reviewed in full and five papers were excluded due to inadequate reporting of methods, for example: sample was not described; no detailed description of data collection and or analysis techniques; the paper reported limited data on breastfeeding; or did not report qualitative data adequately, that is there was no audit trail of the derivation of findings. All studies included in the review had received institutional ethics approval. This resulted in 11 papers being suitable for use in the meta-synthesis (8 qualitative and 3 studies reporting both qualitative and quantitative data) (see Figure 1). Of the 11 papers included, all came from English speaking, high income countries. Only one outlined a theoretical framework; the study by Groleau et al. applied critical theory to analysis and discussion [43]. Two studies indicated that they applied ethnography as a methodology and one described the methodological approach as ethno-nursing methodology [19,29]. The remaining studies, other than those using a survey design, described their studies as either descriptive or interpretive qualitative studies. All the included studies provided an adequate audit trail of the derivation of study findings from the data. We found however, early in the process of analysis that the majority of themes identified in the 11 papers were descriptive in nature and had not been adequately abstracted or conceptualised. This influenced the approach we took to data extraction and synthesis described below.

### Data extraction and synthesis

Data extraction and synthesis was guided by the seven phases used in meta-ethnography articulated by Noblit and Hare [36], which include: identifying the area of interest, deciding what is relevant, reading and re-reading the studies, deciding how the chosen studies are related, translating the studies in relation to one another, synthesising translations and presenting the synthesis [44]. Noblit and Hare [36] suggested that translation involves examining the key concepts in relation to others in the original study and across studies. The way of translating key concepts or interpretive metaphors from one study to another involves an idiomatic rather than a word-for-word translation. Two authors (HO and VS) read each paper and then agreed on the index paper published by Rice and Naksook [19] from which the first set of themes were identified.

The majority of themes reported in the 11 studies were descriptive in nature for example, ‘Reasons for not breastfeeding’ [43]; ‘Health beliefs regarding breastfeeding’ [25] and ‘support for new mothers’ [29]. In four of the 11 papers [14,19,30,45] the authors had included some themes or sub-themes that were more interpretive, most commonly using ‘in vivo’ codes or in a few instances a metaphorical statement. For example, the theme ‘Mother’s milk is best’ identified in this meta-ethnography was developed from themes in the work of Choudhry [14],‘*“Maa ka Dood” – the mother’s milk*‘; Rice [19], ‘Nom reak gerd: beliefs about colostrum’ and Chen [30] ‘purity of breast milk’.

Other authors have similarly reported that the level of abstraction offered in original qualitative research often lacks interpretation [46]. Atkins et al. [46] and Dickson-Swift et al. [47] suggest that to address this some meta-ethnographic studies make use of Schutz’s [48] notion of first, second and third order constructs. First order constructs represent participants views’ or perspectives of the phenomenon under investigation that is, the original quotes that the author’s use to illustrate or represent the theme. Second order constructs, typically used in meta-synthesis, are the thematic statements or abstractions reported as study findings or conclusions by the original authors that should provide more insight or explanation about the phenomenon. In meta-synthesis these themes or second order constructs are then synthesised to produce third order constructs.

Applying this approach the research team worked systematically through the papers, reading and re reading papers to create a list of themes and or metaphors; these were juxtaposed and examined to see how they related to each other [49]. We identified new themes within the first order constructs or original data and where possible we developed second order constructs juxtaposing the authors’ original themes. For example, the theme ‘Producing breast milk requires energy and health’ incorporated authors’ themes such as ‘Mothers' diet and breast feeding’ [19]; ‘Breast is not always best’; experiences of information and role conflict- role conflict’ [14]; and Mothers who bottle feed’ [43] as well as incorporating original data from other authors relevant to this theme. Interpretations by the authors of individual studies were also utilised to ensure the quotes were examined in context.

## Results

Three hundred and twenty-two women who were mothers of children under 5 years of age participated in the 11 studies included in this paper (Table 1). Participants had migrated from a range of countries including China, India, Pakistan, Thailand, Vietnam, Dominican Republic, Puerto Rico and Mexico. The host countries included England, Canada, United States and Australia. Although we did locate studies from non English speaking high income countries, these did not meet the inclusion criteria. In addition, 14 grandmothers of Asian background participated in one of the studies from the UK [29] and 23 white British women were participants in the study by Condon et al. [27]. Participants had been living in the host country for between six months and 29 years (see Table 1). Three studies [14,27,50] also included participants who were born in the host country but who reported that they belonged to a minority ethnic group. The included studies focused on different aspects of migrant women’s experiences of breastfeeding; however, most addressed breastfeeding beliefs, perceptions and experiences as well as infant feeding practices and decisions. The three studies [13,27,45] reporting both qualitative and quantitative data examined the impact of cultural beliefs and practices on infant feeding decisions and practices, and one study [14] examined differences in breastfeeding beliefs and practices based on the level of acculturation.

**Table 1:** Included studies

**Author Country**	**Aim**	**Participants**	**Methodology**	**Methods**	**Results**
Babington [50], United States	· To describe child-feeding practices and Dominican mother’s knowledge and beliefs about healthy size and weight, and obesity.	10 mothers from the Dominican Republic, and 9 mothers from DR but born in the U.S.	Exploratory, descriptive design with thematic analysis	· Focus groups with questions related to breastfeeding, weaning and obesity asked to prompt discussion	· Whilst all mothers saw breastfeeding as beneficial, US born mothers found it to limit freedom
					· Formula rarely used in Dominican Republic due to cost.
					· There were similarities between both groups in regard to introducing foods
Chen [30], Canada	· To explore Chinese mothers’ perceptions about breastfeeding and infant health	15 purposively sampled Chinese mothers two months after delivery in Vancouver Canada Originally came from China, Hong Kong, Taiwan, and South Africa. Living in Canada from 6 months to 29 years	Interpretive qualitative methodology	· Semi structured one to one interview	· Two key themes, 1/ the idea of harmony within change 2/ the meaning of infant health.
				· Data analysed through constant comparison to identify themes	· Breastfeeding viewed as natural and emphasises the importance of the mother’s health for the well-being of infant
Choudhry & Wallace [14], England	· To explore the effect of acculturation on South Asian women and their breastfeeding attitudes and practices	20 South Asian women (11 UK born, 10 born in Pakistan or India), women, who were users of the Children Centres, were of childbearing age, who were expecting or had a child under the age of five and who could communicate in English, Urdu, Hindi or Punjabi. Women assessed as being either low acculturation, bicultural or highly acculturated.	Descriptive qualitative study using thematic analysis	· Semi-structured interviews using a combination of structured and open-ended questions to explore the women’s acculturation status and infant feeding experiences	· Women experienced conflict between traditional and religious breastfeeding practice and UK cultural practices.
					· All women opted for convenient feeding method
					· There was a large family influence in regards to what feeding method was chosen, particularly by the mother-in-law
					· Some women perceive they may be judged for breastfeeding in the UK.
Condon et al. [27], England	· To examine patterns of infant feeding and weaning among ethnic minority groups	Focus groups: 29 women from Bristol from ethnic minority backgrounds. This included women born in Bangladesh, Pakistan and Somalia. One group of Pakistani women and the Afro Caribbean participants were born in UK. Telephone survey: 26 women from Bristol from ethnic minority backgrounds Comparison group: 23 white British women from Bristol	Focus group methodology Thematic analysis	· Focus groups and telephone surveys conducted with women of an ethnic minority background about infant feeding and weaning. Two groups with Pakistani women, one group of women were born in the UK and the other group were born in Pakistan.	· All women in the focus group saw breastfeeding to be the best feeding method, were aware of health benefits and seen as easier. They were also positively encouraged by family and health professionals
				· Quantitative data analysed by SPSS	· Ethnic minority women were more likely to breastfeed.
				· Semi-structured questions analysed using content analysis	
Groleau, Souliere, & Kirmayer [43], Canada	· To identify the cultural factors involved in the abandonment of breastfeeding amongst Vietnamese immigrant women in Canada.	19 immigrant mothers living in Quebec, Canada. Recruited through purposive sampling.	Critical theory and an interpretative approach	· Face to face interviews, interpreters used for 14 women.	· The decision to bottle-feed was not related to acculturation to local practices but to conflicts between Vietnamese cultural practices and the configuration of the new social space in Canada.
					· Living in Canada did not allow specific family members to conduct postnatal traditional rituals thus jeopardizing mothers’ perceived health and the quality of their milk
Ingram et al. [29], England	· To assess South Asian grandmothers’ health beliefs around baby feeding, knowledge of breastfeeding and their ability to support successful breastfeeding	14 Pakistani, Bangladeshi or Indian grandmothers in each focus group	Ethnographic study- focus groups and interviews Thematic analysis	· Topic guide facilitated, tape recorded focus groups and interviews, with demographic questionnaires, to record cultural influence baby feeding. Recordings transcribed and thematically analysed	· All grandmothers felt new mothers need support during postnatal period and see this as their role.
					· Grandmothers believe traditional practices and beliefs, e.g. foods, should be adhered to
					· There is conflict between the beliefs of the grandmothers’ and breastfeeding practices e.g. feeding colostrum
Rice & Naksook [19], Australia	· To examine perceptions, practices and beliefs among Thai women living in Australia in relation to breastfeeding, for the development of a culturally appropriate breastfeeding program in Australia.	30 Thai women living in Melbourne, Victoria who had experienced childbirth in either their homeland, Melbourne or both.	Ethnographic study Thematic analysis	· Interviews with participants in their own home exploring topics around childbearing, childrearing and reproductive health.	· All women believed breast milk to be best for the baby for many reasons e.g. disease prevention. Formula milk was perceived as “animal milk” which could make the child stubborn.
				· Participant observation method to allow observation and recording of women’s cultural beliefs, practices and experiences in Australia.	· Breast feeding seen to have health and relationship benefits for mother
					· The majority of women upheld practices based on traditional beliefs, such as a postnatal diet.
					· There was an apparent conflict between health professionals advice and cultural beliefs
Rossiter & Yam [13], Australia	· To examine Vietnamese women’s perceptions of factors that might influence their choice of infant feeding and how breastfeeding could be promoted and maintained by health professionals	Convenience sample of 124 postnatal Vietnamese women who were born and reared in Vietnam and had a healthy infant born in Sydney in the 6 months before data collection	Survey design collecting quantitative and qualitative data	· Tape-recorded interviews in the women’s homes with semi-structured and open-ended questions which were transcribed verbatim, translated into English and coded.	· The majority of mothers who breastfed knew health benefits of breastfeeding and were supported by significant others
					· The majority of mothers who bottle-fed believed it was more convenient and that Australian formula was as nutritious as breast milk
					· The majority of women experienced difficulties in their interaction with health professionals
Schlickau & Wilson [45], United States	· To explore breastfeeding beliefs, attitudes and practices amongst immigrant Hispanic women in America	Self-identified Hispanic women who participated in a “Moms and Mentors” program, who had recently relocated from Puerto Rico, Mexico and Central or South America.	Ethno-nursing methods - qualitative component. Quantitative data collected to evaluate the efficacy of a model of breastfeeding education.	· Interviews with broad, open-ended statements that allowed for follow-up elaboration and contrast questions in relation to breastfeeding.	· Women knew they would breastfeed and it was seen as easy and convenient.
					· Breastfeeding was common practice in homeland as formula feeding isn’t promoted by hospitals
					· Some women didn’t follow traditional practices due to family influence or necessity.
					· Women liked to be informed.
Straub Melvin and Labbo [51], United States	· To examine Cambodian refugee mothers’ infant feeding beliefs, practices and decision making regarding infant feeding in the US and to explore if a culturally specific breastfeeding program is appropriate for this community.	Convenience sample of 9 women who had come to the US on a refugee visa, had a child between the ages of 6 months and 5 years, had lived in the US for at least 10 years, were at least 18 years old and could speak English.	Exploratory study Thematic analysis	· Self administered questionnaire and a 30minute tape-recorded interview. The tape recordings were transcribed and thematically analysed	· All women continued to practice Cambodian traditions in the US, either food or rituals, as they were not perceived as harmful to the mother or baby
					· All women initiated breastfeeding, but 8 initiated mixed feeding in the hospital due to perceiving they had a low milk supply.
Vaughn et al. [25], United States	· To determined the determinants of breastfeeding for Latina mothers in the Cincinnati area	40 Latina mothers, aged over 18years, who had babies under 4 months, were foreign born, had lived in America for less than 10 years and had tried breastfeeding at least once.	Survey design collecting quantitative and qualitative data Constant comparison analysis based on grounded theory approach	· Semi-structured interview and a validated *Breastfeeding Self-efficiency Scale* questionnaire.	· Majority of mothers planned to breastfeed for at least one month and were receiving education
					· Women wanted language specific breastfeeding education and support was seen as a major influencing factor.

Through the process of reciprocal translation, one overarching theme emerged: ‘Breastfeeding in a new country: facing contradictions and conflict’. This theme comprised four sub-themes: ‘Mother’s milk is best’; ‘Contradictions and conflict in breastfeeding practices’; ‘Producing breast milk requires energy and good health’; and ‘The dominant role of female relatives’ (see Table 2 for themes arising out of included studies). Contradictions and conflict were evident through each sub-theme and occurred within and across cultures. Women’s traditional beliefs and practices were often different from those they observed in their new social context and what they learnt from health professionals in the host country. For some women, contradictions and conflict occurred when family and friends took on the dominant cultural practices of the host country and the new mother was somewhat torn between her own more traditional beliefs and those of key family members such as the mother-in-law [14,27,29]. In contrast, other women maintained traditional practices, often ignoring messages from health professionals in the host country [19,30]. Contradictions and conflict resulted in uncertainty and anxiety in new mothers, particularly if mothers were unable to practice the traditional postpartum rituals of their native countries, often resulting in the perception of insufficient breast milk [14,43,45].

**Table 2:** Sub themes in each qualitative paper

	**Mothers’ milk is best**	**Contradictions and conflict in breastfeeding practices**	**Producing breast milk requires energy and good health**	**The dominant role of female relatives**
Babington, [50]. United States.	√			√
Chen, [30]. Canada.	√		√	√
Choudhry & Wallace, [14]. UK.	√		√	√
Condon et al. [27]. England.	√	√	√	√
Groleau, Souliere, & Kirmayer, [43]. Canada.	√		√	√
Ingram, Johnson & Hamid, [29]. England.	√	√	√	√
Rice & Naksook, [19]. Australia.	√	√	√	√
Rossiter & Yam, [13]. Australia	√	√	√	√
Schlickau & Wilson, [45]. USA	√	√	√	√
Straub Melvin & Labbok [51]. USA.	√		√	
Vaughn et al. [25]. USA	√		√	√

### Mothers’ milk is best

In almost all of the included studies there was consensus from participants that mother’s milk is best for the baby. In some studies women emphasised the health benefits, citing examples of information received from family [19,30,43] or related to common cultural beliefs in their country of origin. For example, one Thai woman living in Australia stated, ‘people in Thailand say that the mother’s milk has a lot of immunity, and that is why the baby does not become ill easily’ [19], p 14]. In many studies, breastfeeding was described as the ‘natural’ way to feed a baby [30]. Chinese women in Canada, for example, saw breastfeeding as part of ‘natural or dynamic laws by which humans live…the process of change that reflects and influences both a mothers’ health and that of her baby…’ [30], p. 1023].

In the majority of studies, migrant women took the decision to breastfeed for granted. For example, Latino women living in the United States [45], p. 27] stated, ‘I never doubted [breastfeeding]’, ‘you just know you will breastfeed’, and ‘it's the way it's supposed to be’. For Chinese women living in Canada, breastfeeding was part of the ‘natural work’ of a mother. As one woman stated, ‘the crucial thing I give is devotion, my love for my baby. I perform a mother’s duty…I do not think about nutrition and I know little about it’ [30], p. 1023]. These women were surprised that so many women in their host country did not breastfeed, ‘I don’t understand…after delivery, they directly formula fed their baby. They told me they do not have breast milk’ [30], p. 1024].

Breastfeeding was also important to mothers for the mother-child relationship [19,27,30]. In many of the studies, women described breastfeeding as ‘a gift’ to the child and as ‘…a beautiful feeling to see him look at me from my breast…It is the most peaceful rewarding moment in my life‘ [27], p.346], and one woman felt that with breastfeeding, ‘(the baby has) more ‘tendresse’ to you. It is the best relationship, the baby likes you so much, only wants the mother’ [27], p. 346]. Giving breast milk was likened by Thai women in Australia [19] and Pakistani grandmothers in the UK [29] to sharing one’s blood with the infant:

…breast milk comes from our blood. The blood changes into milk. My mother told me about this. She used to say to me that ‘Do you know that every drop of milk that you take is from my blood’ and she would ask me if I love her. I am now teaching my kids this belief too. I am very proud of this close relationship between my kids and me [19], p. 15].

So important was the breastfeeding relationship with the child that one Latino mother decided to formula feed after birth as she was to return to work. She spoke of not wanting to breastfeed so that her child would not feel deprived, ‘it is better to give formula quickly so that the baby doesn’t suffer when you have to leave them to be cared for by another person’ [25], p. 323].

For many mothers in the included studies, breastfeeding was also associated with the positive development of the baby’s personality [14,19,25,29]. Women stated that breastfeeding ’deliver(s) goodness and mak(es) the baby grow up to be a good person’ [14], p.79], and ‘oh he/she’s like that because he had the mother’s milk. . . such as the way he develops his personality or intelligence’ [14], p.79]. As one Thai woman in Rice’s Australian study described, ‘in my hometown, people say a bottle-fed infant will be stubborn because he or she is fed with cow milk, not human milk’ [19], p. 14].

Only in two studies was formula milk described by some participants as equal to, or better than, breast milk [13,14]. In the UK, Choudhry and Wallace reported ambivalence among women from countries such as India, Pakistan and Bangladesh about the value of breast milk and breastfeeding [14]. In this study, women spoke about the infant feeding practices they had observed, for example, ‘in Pakistan most people would breastfeed but here (referring to the UK) everyone bottle feeds’ [14], p. 81] and another added:

I thought if they’re (peers from the Anglo Culture) doing it and there’s no harm then there’s no need for me to go through the pain of trying to breastfeed when I could give them formula [14], p. 81].

Some women in Choudhry and Wallace’s study perceived their breast milk to be inadequate or less nutritious, a message promoted by some family members. In four studies women reported that formula feeding was more convenient, offering more freedom to ‘get on with our lives’ [50], p. 392], an easier option and a way to share the responsibility of infant care [14], a way to manage the daily hassles and stresses of life with a baby in a new country with limited support and at times, to address breastfeeding difficulties [13,14,25]. It was rare however, for women in these studies to report experiencing pain with breastfeeding. Pain was only reported in three studies and was not directly related to participant’s personal experiences [14,19,25]. For example, one woman living in the US stated, ‘I think women give formula because of the pain, your breasts can suddenly hurt a lot when breastfeeding’ [25], p.324]. Some women also reported formula feeding if they had to return to work, ‘I was unable to breastfeed for a longer period because I had to work after childbirth to assist with family finances’ [13], p.274].

Two studies highlighted the implications of visibility and affordability of infant formula in the host countries in this case, Australia and the US [13,25]. Latino women in Vaughan’s study observed that:

In Mexico it would be more likely that you would give your child breast milk, compared to here; simply because there the milk (formula) is much more expensive and it is not like here where the government gives out milk (formula) [25], p. 324].

### Contradictions and conflict in breastfeeding practices

In many of the included studies, migrant women talked about receiving mixed messages about different breastfeeding practices, such as giving colostrum, length of a breastfeed, age at which breastfeeding should cease and breastfeeding in public. At times contradictions or conflict were easily resolved and women felt comfortable to maintain their traditional breastfeeding practices whilst at other times this conflict resulted in heightened confusion or distress.

#### Colostrum: perfect food or best discarded

Only four studies [19,25,27,29] reported women’s perspectives on feeding a newborn colostrum and the majority agreed with this practice. Contradictory perspectives on colostrum tended to occur within cultures rather than between cultures. For example, some Thai women in Australia emphasised the value of colostrum reporting that ‘old people in Thailand say the first milk is very good for the baby because it will help to clean inside the baby’s body’ [19], p.15]. Alternatively, one study also found some women were taught by their mothers to ‘squeeze it out’ as it is not ‘real’ milk and it is not clean’ [19], p.16]. The potential for intergenerational conflict around colostrum was highlighted in Ingram’s study of Pakistani grandmothers in the UK [29]. They found that despite health campaigns about the health benefits of colostrum in low and middle income countries such as India and Pakistan, these grandmothers emphasised to their daughters or daughter-in-law living in the UK that colostrum should be discarded as it is, ‘old and had been stored in the breast for a long time’ [29], p.322].

The decision to give colostrum was also influenced by instructions from health professionals in the new culture [19]. Rice and Naksook reported that some Thai women in Australia privileged new knowledge gained from health professionals over traditional practices stating:

in my hometown people believe that colostrum is not good for the baby because it is like infectious stuff’…I am doing what they say here (in Australia), if you believe too much of your own tradition, it may not be good for your child [19], p.16].

#### Breastfeeding: for how long and how frequently?

Given the positive views towards breastfeeding expressed by migrant women, it is not surprising that some participants emphasised the importance of maintaining breastfeeding well into the second year of life. Thai women spoke of breastfeeding as long as possible, for example, one woman stated, ‘in my village in Thailand mothers keep feeding until they are about to give birth to another child’ [19], p.17]. Alternatively, negative perceptions of this practice from others may result in cessation:

(I) stopped breastfeeding because it was time to do so. In fact I didn’t want to stop it and my husband didn’t either... But my friends kept teasing me. They said ‘Look at your boy, his legs are hanging down to the floor already [meaning he is too old to be on her breasts] and you still suckle him’…So I felt embarrassed and decided to stop him [19], p.18].

Religious beliefs also influenced breastfeeding practices [14,27,29]. For example, South Asian grandmothers emphasised, ‘the Koran states that mothers should feed for two or two and a half years and mother’s milk stays in the child’s body for 40 years' and ‘Allah gives milk to the breast as a gift for the baby’ [29], p.322]. The religious teachings were also a significant source of tension for some women who felt pressured by older relatives to continue breastfeeding beyond six months [27].

The practice of ‘on demand’ or scheduled feeding may also create conflict between cultural beliefs and dominant views in the host culture, including the perceptions of health professionals. In the following quotes, Thai women describe the tension they experienced with health professionals:

…the doctor here told me that I should not spoil my baby by feeding her any time she wants and I should feed her on schedule…But I don't listen to the doctor. Whenever my baby wants it, I just feed her [19], p.16].

#### Where to breastfeed?

Feeling embarrassed by breastfeeding when others are present in the home or in public spaces was discussed by some migrant women as a reason to formula feed or to use mixed feeding. Latino mothers in the US emphasised that breastfeeding in public made them feel uncomfortable. As one woman stated, ‘the most difficult thing is going out in public…it embarrasses you to breast-feed in public and I don’t feel comfortable…Right now I am not going out in public, but later on when I do, I will give him formula’ [25] p.324]. Women in Choudhry and Wallace’s study also highlighted this contradiction, ‘religion teaches breastfeeding best (in home country) but in [this] culture (in the UK) [it is] taught that formula feeding is the most convenient, because there is less embarrassment for the mum’ [14], p. 79]. Similarly, some women in the study by Condon et al. reported, ‘…they might judge me or something (for feeding in public) because it is something we do at home’ [27], p.81].

### Producing breast milk requires energy and health

In eight of the 11 studies women emphasised the importance of adhering to traditional postpartum practices related to diet, rest and body care to ensure that their body had the energy to produce enough breast milk. Women in Chen’s [30] and Groleau et al.’s [43] studies who had migrated to Canada prioritised care for the maternal body over specific practices related to caring for the infant including feeding. Stated simply by Latino women in the US, ‘if you are healthy, the baby is healthy’ [45], p. 28]. The importance of diet and rest was expressed by many of the mothers. As one woman in the study by Condon et al. in the UK explained, ‘you need well prepared food, time to feed and rest yourself, if you don’t have these you won’t be able to produce enough milk for the baby’ [27], p.346]. Chen reported the importance of diet for Chinese women stating:

In traditional Chinese culture, the mother’s body is weak after delivery. She must have tonic soup (Chinese herb soup) in order to recover her vitality and energy… Then the breast milk naturally comes out [30], p. 1024].

Study participants also emphasised the importance of consuming *hot* foods, resting and exposing their body to ritualised steam baths, massages and having a constant source of *heat* under the bed (for example, a pot of burning charcoal or an electric blanket) so that the body of the postpartum mother will recover, closing pores and reconstituting the lost blood and *heat* and thus regain the strength and equilibrium necessary for health [19,30,43]. This was most evident in the studies that included women who were born in Vietnam, Thailand and China [13,19,30,43]. As Chen described,

During this period, after eating the tonic soups [prepared by family members], I had a lot of breast milk. When my first baby sucked my breast on this side, milk naturally flowed out from the other breast [30], p. 1025].

The importance of women resting for at least one month in the postnatal period was emphasised. Respondents in Groleau et al.’s study explained that to maintain heat ‘mothers are given a traditional steam bath twice a day usually performed by an elderly woman in her family, usually her own mother with ingredients such as lemon, guava or grapefruit’ [43], p.520]. This ritualised ‘time out’ lasts longer for primiparous mothers (100 days) as opposed to one month for multiparous mothers. According to Vietnamese tradition, breastfeeding is contraindicated for women experiencing postpartum fatigue, because the production of milk requires a lot of ‘vital energy’ [43], p.520].

Women in many of the included studies reported becoming anxious if they could not adhere to traditional practices [13,19,27,29,45]. For example, in the study by Groleau et al. [43], none of the Vietnamese women were exclusively breastfeeding and only those who were multiparous initiated breastfeeding. Groleau et al. [43] reports that initially women explained they were not breastfeeding because they had to return to work or that they did not have enough milk yet; however, when probing deeper, the women spoke about their concern about losing heat from their bodies because they were not participating in traditional postpartum practices. In contrast, the Latino women in Schlickau and Wilson’s study were somewhat ambivalent about these practices. While acknowledging the traditional ways of keeping healthy, they simultaneously faced the problem of returning to work. As one woman stated, ‘I was on the farm with the cows and the children and could not stay in bed for 40 days…You have to take care of yourself, though, during that time’ [45], p.28].

As a consequence, some women prepared for insufficient milk, ‘you prepare for not having enough milk, so you buy bottles and powdered milk’ [27], p.346] and others reported that they were more likely to bottle feed:

A mother’s body is the foundation of breastfeeding. I breastfed my previous child but this time I fed formula to this baby because my body was very weak after pregnancy. I also did not receive good quality Chinese traditional postpartum care [30], p. 1025].

I only breast fed my baby for two months because I was concerned about my body and due to this I did not produce enough milk for her..I do not eat those things that people say will help to make a lot of milk, so my milk was diminishing’ [19], p.18].

Messages from others including health professionals can also result in the mother assuming she won’t have enough milk. This was evident in Straub, Melvin and Labbok’s (2001) study in the US when a Cambodian woman stated:

I think the nurse brought it, the formula. And they asked me [if] I want to breastfeed and I said yes, she asked if I wanted formula and I said yes…Well, I was thinking that maybe I wasn’t producing enough milk…I just didn’t know if I had enough [51].

### The dominant role of female relatives

In most of the studies the importance of being cared for by family was emphasised. In three studies [14,19,25] women described the role that their partners played in supporting breastfeeding. Other women indicated that their partners were not involved and indeed were often a hindrance [14,25]. Most support came from the woman’s own mother and or mother-in-law. As one participant in Chen’s study described:

Babies are bigger and stronger in China. They look healthier. Probably in China a baby eats well [and has good quality of breast milk from mother] and [also] has good quality of care from the grandmother, grandmother-in-law. But in Canada the babies do not look healthier and stronger; they look slim. Westerners do not spend a lot of time taking care of their children and they do not have many family members to help them [30], p.1026].

This was also well illustrated in Groleau et al.’s [43] study where women who had previously given birth in refugee camps spoke of the support they received from other female relatives or community members who were living with them in Vietnam. However, this was not their experience in Canada. Groleau et al. [43] found that the constraints of living in a new culture brought about by the absence of close family and the support it normally provides meant that many Vietnamese women decided to bottle-feed rather than breastfeed. This was seen as the best option for preserving their own and their baby’s health. As one woman in Groleau et al.’s study describes:

In Vietnam, she received a lot of visitors [after the birth]. In the Philippines [in the refugee camp], she received family members. In Canada, she doesn’t receive anyone; she is all alone with her husband [43], p.521].

The decision to formula feed appeared to be influenced by views of family members living in the new country, such as her mother-in-law. This is also well illustrated in Choudhry and Wallace’s study, where women living in the UK made statements such as, ‘my mother-in-law encouraged bottle feeding for my own benefit, I was able to share the feeding with her which was good for me’ [14], p.79] and:

I only breastfed for a while, because when I started I realised that my mother in law was right, I wasn’t able to cope with everything myself. Formula-feeding meant that whilst I was doing other work, my mother in law was able to help out and feed my baby [14], p.80].

These persuasive views of a mother-in-law may also contradict views of the woman’s own mother who may be still living in the country of origin. One mother in Choudhry and Wallace’s study in the UK explained, ‘When I lived in Pakistan my mum always encouraged me to breastfeed my baby but after I got married and came here I was told something different by my mother in law’ [14], p.80]. Another woman stated, ‘my parents advised that I should breastfeed but I found it really hard to fit into my life and the things I have to do around the house, like housework and looking after my mother in law’ [14], p.80].

## Discussion

In this meta-ethnographic study we have synthesised the findings of 11 studies reporting qualitative data on the breastfeeding experiences and practices of migrant and refugee women now living in a high income, English speaking host country. The overarching theme, ‘Breastfeeding in a new country: contradictions and conflict’, represented not only a clash between an individual woman’s beliefs and practices and the dominant practices in the new country, but also a tension with family members either in the country of origin or in the new country. This conflict is at times exacerbated by a woman’s own expectations as a mother and her material circumstances [43].

Breast milk and breastfeeding were afforded high status by women from diverse cultures, with only two studies finding some women perceived formula milk to be equivalent to breast milk. This is reflected in the higher breastfeeding initiation and duration rates amongst migrant women compared to native born women in high income countries [5,7,16]. Only four studies included beliefs about colostrum and for the most part these were positive, contradicting a common assumption among health professionals that many women from ‘Asia’ do not offer colostrum [20]. Although some participants provided reasons for why they or other women may decide to feed with formula, overall, there was a view that ‘breast is best’ and that breastfeeding is the ‘natural way’ to feed a baby. It is interesting to note that we excluded a number of papers from this synthesis because they did not report substantial qualitative data related to breastfeeding [52,53]. We suggest that one explanation for the limited data on breastfeeding in these infant feeding studies may be because participants indicated there was little to discuss as breastfeeding was ‘what you do’, they planned to do it, thought it was best for the baby and did not elaborate during interviews with the researchers [53].

It appears that participants from a range of cultural backgrounds living in the UK were less positive towards breastfeeding, for example, the women from Pakistan who participated in Choudhry and Wallace’s study. Similar views reflecting negative attitudes towards breastfeeding in western cultures have been reported among Chinese and Vietnamese women in Australia and Ireland, with some seeing breastfeeding as inconvenient, embarrassing and leading to dependency in the child [10,12,13,28,54].

Despite the positive view of breastfeeding and the importance of breast milk for infants, the research findings suggest that migrant and refugee women can struggle to continue breastfeeding while managing life with a new baby in a new country. Some migrant and refugee women will be poorly resourced, have to return early to paid work, speak little English, and have responsibility to maintain the house without their mothers, mothers-in-law and other family members to support them. In these circumstances, some women indicated that it was best not to even start breastfeeding [25,43], particularly if important postpartum cultural practices that maintain health and energy cannot be adhered to.

Most of the studies reported on the importance of a variety of postpartum rituals for the adequate production of breast milk. Groleau et al. argue that the ritualised exposure of mothers to heating and other practices are ‘the cornerstone of a rite of passage to motherhood, a key moment for primiparas to acquire their new identity as mothers’ [43], p. 520]. If a woman wishes to maintain these practices but is unable to, she may become anxious about her ability to produce sufficient milk. This is more likely to result in cessation of breastfeeding [13,28,43]. This is not because the woman does not value breast milk, rather, as Groleau and colleagues suggest, it is her own milk that she does not consider of high value [43]. The concept of insufficient milk is also common in western cultures [55,56] with many studies exposing the profound mistrust that women have in their bodies and the lack of confidence in their capacity to breastfeed [57-59].

The majority of included studies did not examine differences in breastfeeding practices between women who were recent arrivals and those who had lived in the host country for many years. There were two exceptions [14,45]; Choudhry and Wallace found that women who had low levels of acculturation 'were not influenced by the new culture they were living in, and continued to breastfeed their infants as directed by South Asian cultural teachings about the psychological benefits of breastfeeding' [14], p. 82]. When these women did opt to formula feed, it was in response to conflict they experienced either between the information they received about the best form of feeding or between their roles as a mother and daughter-in-law. Opting to formula feed was a way to resolve the conflict [14]. These experiences are reflected in some studies of the impact of acculturation. A large cohort study in the US reported that for every year that a migrant woman lived in the US, her odds of breastfeeding declined by 4% and, her odds of doing so for at least six months, declined by 3% [16].

In contrast, Grewal, Bhagat and Balneaves [52] report on an apparent resurgence in second and third generation Punjabi families living in Canada assuming traditional practices related to birth and parenting. Together with Groleau and colleagues, Grewal, Bhagat and Balneaves [52] caution against the blind acceptance of the acculturation hypothesis and warn health professionals not to make assumptions about perinatal preferences based on time since immigration. Furthermore, Groleau, Soulière and Kirmayer. [43], p. 542] argue that ‘the acculturation thesis emphasises the role of the host culture in influencing practices but does not pay any regard to women's agency.’ They believe that beliefs and practices linked to breast milk are dependent on, and embedded in everyday cultural practices. This cultural knowledge is ‘maintained and expressed not only through explicit knowledge or beliefs but also in practices that depend on a specific configuration of social space' [43], p. 524] that is, the everyday living circumstances of women.

The final sub-theme reflects the central place of female family members in the lives of new mothers. Female family members who are available to assist a new mother to participate in the traditional postpartum practices of their native countries were considered by migrant mothers to be important in the maintenance of breastfeeding. Alternatively, through migration or refugee status, some new mothers lacked family support [43] or the support offered by family members led to conflict and tension [14], increasing the likelihood that the new mother would cease breastfeeding. For example, in the study by Choudhry and Wallace [14], some women reported that their role as a daughter in law, and the associated expectations, meant that it was difficult to accommodate breastfeeding in her life as a mother. A recent population based study of the feeding practices of migrant women in the US found that their parents’ country of birth was a significant predictor of breastfeeding initiation and duration [7]. This highlights not only the importance of understanding the cultural beliefs and practices of new mothers but also those of their mothers and mothers-in-law.

In the absence of a supportive network, women may turn to health professionals for advice. Few of the 11 included studies reported on women’s experiences with health services and health professionals and what was reported tended to be negative. Other studies have reported the barriers that migrant and refugee women experience when seeking services, including language barriers, experiences of discrimination, and conflicting belief systems [52]. It also appears that some health professionals report it is easier to provide care to women from ethnic minorities born in the host country rather than recently arrived migrant women, citing language barriers as a significant impediment [35]. Research conducted by McFadden, Renfrew and Atkin [34] revealed a level of apathy amongst maternity care professionals towards supporting migrant women with breastfeeding. This apathy was fuelled by the belief that migrant women disregarded professional advice in favour of the advice from family, especially grandmothers. The dominant assumption amongst a majority of health professionals was that migrant women were too ‘submissive’ to the influence of their family [34]. We argue, along with Puthussery et al., [35] that incorrect assumptions and stereotyping by maternity care providers can lead to attitudes and behaviours that reflect subtle forms of ‘institutionalised racism’ which require innovative intervention strategies to overcome.

### Study Limitations

The findings of this meta-ethnographic study are limited by the nature and depth of data collection and analysis in each study. As discussed, most studies were descriptive in nature, and did not identify metaphorical statements in their original work. This made it difficult to conduct reciprocal translation. To facilitate the synthesis we adopted the approach described by Atkins [46] working with first order constructs presented in the papers.

It is also important to emphasise that in this meta-ethnography we have synthesised studies reporting the experiences of women who come from diverse cultural backgrounds and it is inappropriate to attempt to create one picture of migrant and refugee women's breastfeeding experiences and practices. We also note that there are inconsistencies in the approach and terminology used in the original papers when identifying and categorising the ethnicity or cultural background of participants. In some studies, the ethnic background of participants was only identified by country of birth [13,45]. Alternatively, some researchers asked participants to identify their ethnic or cultural background when completing a demographic questionnaire but it is not clear if these were open ended or pre categorised responses [25]. Two studies [14,27] explicitly stated that women were asked to self identify their cultural background or ethnicity. Others made no mention of how these details were obtained [30,43,50]. Commentators have emphasised the complexity of assigning ethnicity to study participants [40,41] indicating a preference for the value of self assigned ethnicity, although recognising that this adds a level of complexity to data collection and interpretation. This complexity often results in researchers using inappropriate labels to describe cultural background. For example, the terms Asian and South Asian were used in a number of papers to describe people from the Indian sub-continent, many of whom, if asked, would vary in how they described their ethnic identity.

## Conclusion

Although the WHO recommends exclusive breastfeeding to six months [1], studies report mixed findings regarding the rates of both exclusive and partial breastfeeding amongst women who are migrants and refugees in high income countries. From the findings of this meta-ethnographic study it appears that migrant women experience numerous challenges to breastfeeding in a new country.

To support migrant and refugee women to breastfeed, health services need to provide access to culturally appropriate care with adequate interpreting services and health professionals who have had training in cultural competence. Women may benefit from access to a range of health education materials in diverse languages in printed form, in DVDs or online. But this is not a substitute for midwives and other professionals being sensitive and taking the time to listen to women’s needs.

This meta-ethnographic study indicates that health professionals do not need to convince migrant and refugee women of the importance of breastfeeding, there are however, areas where women may need accurate information; for example, related to perceptions of insufficient breast milk. Strategies to engage grandmothers in educational opportunities are also particularly important. Facilitating opportunities for women to receive support from peers has demonstrated success in some studies [32,45]. Choudhry and Wallace [14] also suggest that if migrant women had the opportunity to observe peers from the dominant Anglo culture engaging in breastfeeding, it may help redress the misconceptions that British women do not breastfeed. There may also be value in establishing midwifery continuity of care models for migrant and refugee women to build their confidence with health services. Finally, we emphasise the importance of ongoing education for health professionals to increase awareness of the diversity that exists in breastfeeding beliefs, practices and experiences within and across cultures.

The findings of this meta-ethnographic analysis indicate areas for further research. For example, a greater understanding of the diversity of infant feeding beliefs and how women negotiate practices including the giving of colostrum, adhering or not to traditional postpartum practices and management of early return to work may inform understanding of how women form an identity as a migrant woman who has transitioned to motherhood in a new country. It is also important to study health professionals' attitudes towards migrant women's breastfeeding ability, discomfort about breastfeeding in public, the role of the mother-in-law, and duties to the extended family. In the studies included in this meta-ethnography, few women reported experiences of pain associated with breastfeeding in contrast to many studies conducted in the host countries where pain is the prime reason for breastfeeding cessation. This warrants further study. The visibility of infant formula in the host countries is concerning and research into the affordability and visibility of infant formula from the perspective of women recently arrived in the host country may help understand the subtle and pervasive influence of socio cultural expectations and practices.

## Competing interests

The authors declare that they have no competing interest in the present research.

## Authors’ contributions

VS, EB and HD: participated in the study design, HO conducted the search and extracted themes and data from each paper and prepared the summary tables, EB prepared the methods section of the first draft. All authors participated in the analysis and drafted the manuscript. All authors approved the final version.

## Pre-publication history

The pre-publication history for this paper can be accessed here:

http://www.biomedcentral.com/1471-2393/12/163/prepub
